# Using Social Media for Actionable Disease Surveillance and Outbreak Management: A Systematic Literature Review

**DOI:** 10.1371/journal.pone.0139701

**Published:** 2015-10-05

**Authors:** Lauren E. Charles-Smith, Tera L. Reynolds, Mark A. Cameron, Mike Conway, Eric H. Y. Lau, Jennifer M. Olsen, Julie A. Pavlin, Mika Shigematsu, Laura C. Streichert, Katie J. Suda, Courtney D. Corley

**Affiliations:** 1 Data Sciences and Analytics Group, Pacific Northwest National Laboratory, Richland, Washington, United States of America; 2 International Society for Disease Surveillance, Boston, Massachusetts, United States of America; 3 Commonwealth Scientific and Industrial Research Organization Digital Productivity Flagship, Canberra, Australia; 4 Department of Biomedical Informatics, University of Utah, Salt Lake City, Utah, United States of America; 5 School of Public Health, The University of Hong Kong, Hong Kong Special Administrative Region, People’s Republic of China; 6 Skoll Global Threats Fund, San Francisco, California, United States of America; 7 Henry M. Jackson Foundation for the Advancement of Military Medicine, Bethesda, Maryland, United States of America; 8 National Institute of Infectious Diseases, Shinjuku-Ku, Tokyo, Japan; 9 Center of Innovation for Complex Chronic Healthcare, United States Department of Veterans Affairs, Hines, Illinois, United States of America; IFIMAR, UNMdP-CONICET, ARGENTINA

## Abstract

**Objective:**

Research studies show that social media may be valuable tools in the disease surveillance toolkit used for improving public health professionals’ ability to detect disease outbreaks faster than traditional methods and to enhance outbreak response. A social media work group, consisting of surveillance practitioners, academic researchers, and other subject matter experts convened by the International Society for Disease Surveillance, conducted a systematic primary literature review using the PRISMA framework to identify research, published through February 2013, answering either of the following questions:

1. Can social media be integrated into disease surveillance practice and outbreak management to support and improve public health?
2. Can social media be used to effectively target populations, specifically vulnerable populations, to test an intervention and interact with a community to improve health outcomes?

Examples of social media included are Facebook, MySpace, microblogs (e.g., Twitter), blogs, and discussion forums. For Question 1, 33 manuscripts were identified, starting in 2009 with topics on Influenza-like Illnesses (n = 15), Infectious Diseases (n = 6), Non-infectious Diseases (n = 4), Medication and Vaccines (n = 3), and Other (n = 5). For Question 2, 32 manuscripts were identified, the first in 2000 with topics on Health Risk Behaviors (n = 10), Infectious Diseases (n = 3), Non-infectious Diseases (n = 9), and Other (n = 10).

**Conclusions:**

The literature on the use of social media to support public health practice has identified many gaps and biases in current knowledge. Despite the potential for success identified in exploratory studies, there are limited studies on interventions and little use of social media in practice. However, information gleaned from the articles demonstrates the effectiveness of social media in supporting and improving public health and in identifying target populations for intervention. A primary recommendation resulting from the review is to identify opportunities that enable public health professionals to integrate social media analytics into disease surveillance and outbreak management practice.

## Introduction

Social media communication is an increasingly utilized outlet for people to freely create and post information that is disseminated and consumed worldwide through the Internet. News media, traditional scientific outlets, and social media create a platform for minority viewpoints and personal information, which is not being captured by other sources. Social media can create a sense of anonymity, allowing for unadulterated personal expression when compared to traditional face-to-face meetings, especially among young people and about intimate matters [1]. In this respect, social media provide an additional informal source of data that can be used to identify health information not reported to medical officials or health departments and to reveal viewpoints on health-related topics, especially of a sensitive nature.

In the past 10 years, research articles connecting disease surveillance with Internet use have increased in number, most likely due to the increase in availability of health-related information from various Internet sites. For example, Wikipedia article hits [2], Google search terms (Google Flu Trends) [3], and online restaurant reservation availability (OpenTable) [4] were modeled against the number of patients with influenza-like illness (ILI) reported by the Centers for Disease Control and Prevention (CDC). Several literature reviews have looked at the potential of this type of research to benefit human health.

Moorhead et al. conducted a review of research studies to identify potential uses, benefits, and limitations of social media to engage the general public, patients, and health professionals in health communication [5]. Although articles identified benefit from using social media in health communications, the authors note a lack of research focused on the evaluation of short- and long-term impacts on health communication practices. Bernardo et al. provided a scoping review of the use of search queries and social media in disease surveillance [6]. First reported in 2006, the reviewed literature highlighted accuracy, speed, and cost performance that was comparable to existing disease surveillance systems and recommended the use of social media programs to support those systems.

Velasco et al. defined their literature review to contain only peer-reviewed articles on event-based disease surveillance [7] in which they identified and described 12 existing systems. Walters et al. described numerous systems implemented and dedicated to biosurveillance, defined as “the discipline in which diverse data streams such as these are characterized in real or near-real time to provide early warning and situational awareness of events affecting human, plant, and animal health,” many of which center around human disease outbreaks [8]. The paper points out that including emerging media, such as blogs and Short Message Service (SMS), into these systems along with standardized metrics to evaluate the performance of different surveillance systems is crucial to the advancement of these early warning systems.

As members of the International Society for Disease Surveillance (ISDS), we established a social media working group (henceforth called the workgroup) to develop research, technology, and operational innovations in electronic public health surveillance. We proposed to evaluate the use of social media to enable public health professionals to realize positive, valuable, and timely community health outcomes at the local, state, regional, national, and global levels. To address these goals, we followed the PRISMA process [9] by systematically compiling and analyzing literature that demonstrates innovation in electronic public health surveillance through the use of social media.

By focusing on how research on social media data (further defined below) can be used for actionable disease surveillance, we are able to bring to light the best ways of using these tools to target vulnerable populations and improve public health in the broad spectrum from identifying and monitoring disease outbreaks to addressing traditionally intractable health concerns, such as adolescent drug and alcohol use.

## Methods

This systematic review builds upon the preferred reporting items outlined in the PRISMA Statement in effort to properly assess the quality and quantity of health-related research using social media analytics for active surveillance, S1 Checklist. A social media application was defined for this review as, “an Internet-based application where people can communicate and share resources and information, and where users can activate and set their own profiles, have the ability to develop and update them constantly, and have the opportunity to make such profiles totally or partially public and linked with other profiles in a network.” Examples of social media included in this review are Facebook, MySpace, microblogs (e.g., Twitter), blogs, and discussion forums. Articles using data sources, such as Internet searches, ProMed-mail, and citizen-generated data were not included. In March 2013, a query of scientific literature databases (PubMed, Embase, Scopus, and Ichushi-Web) was conducted for all literature published through February 2013 to determine potential publications for review by the workgroup (Table 1).

**Table 1:** The databases (PubMed, Embase, Scopus, and Ichushi Web) that were queried and the search terms applied to identify potential publications for review.

Database	Type of Search	Search Terms
PubMed database (http://www.ncbi.nlm.nih.gov/pubmed/)	Medical Subject Headings (MeSH)	“Internet,” “social media,” “blogging,” “biosurveillance,” “disease outbreaks,” “epidemics,” “communicable diseases,” “population surveillance,” “sentinel surveillance,” “public health”
Embase database (http://www.elsevier.com/online-tools/embase)	Emtree (Elsevier Life Science thesaurus)	“social media,” “Internet,” “social network,” combined with “biosurveillance,” “epidemic,” “pandemic influenza”, “pandemic,” “infection,” “communicable disease,” “outbreak”
Scopus (http://www.scopus.com/)	General terms	Combination of PubMed and Embase search terms
Ichushi-Web (http://www.jamas.or.jp/personal_web/login.html)	General terms	Japanese translated combination of PubMed and Embase search terms

Searches were further refined to include only human subjects and to exclude review (i.e., meta-analysis or other systematic reviews) and editorial articles. Articles published in Italian, German, Dutch, English, Spanish, and Japanese were included in the search check box because of multilingualism within the workgroup. In addition to these searches, other articles reviewed for potential inclusion were the ISDS research committee monthly literature review collection (http://www.syndromic.org/cop/research) and references from relevant articles, systematic reviews, and meta-analyses found through initial literature searches. The online bibliographic service Zotero (https://www.zotero.org/) was used for citation management.

The workgroup was formed from members of the ISDS with diverse background specialties, (e.g., public health physician, doctor of veterinary medicine, data scientist, public health professor, biomedical informatics) and countries of residence (e.g., USA, Australia, China, Japan). Within the group, a pair of members evaluated each collected abstract in detail for possible inclusion in the systematic review. Each member recorded the following information from each potential publication: author(s), date of publication, publication type (e.g., journal, conference proceedings, white or gray literature), data source type (e.g., social networking sites, microblogs, or open source databases). Requirements were that each study must be published as original researchand must analyze social media. The initial review was done for all documents containing an abstract, including peer-reviewed conference proceedings or white papers. An article was excluded if the full text was not available, if only methods were described (i.e., building an application programming interface, but no results), or if it did not directly address one of the two following research questions:

1. Q1. Can social media be integrated into disease surveillance practice and outbreak management to support and improve public health?
2. Q2. Can social media be used to effectively target populations, specifically vulnerable populations, to test an intervention and interact with a community to improve health outcomes?

Any differences of opinion about whether to include a paper were resolved through discussion until the workgroup achieved a consensus on inclusion or exclusion.

For each article fitting the review inclusion criteria, one workgroup member was assigned to extract and record specific details from the full-text article. This information included background (e.g., study objective, sample population and size, and the location, setting, time, and duration of the study), methods (e.g., study design, keywords, classification methods), outcomes measured (e.g., population, disease studied, intervention or exploratory (i.e., whether the study evaluates the impact of or observes the use of social media, respectively), hypothesis, outcomes related to either research question), results, and conclusions. To assess involvement of a public health jurisdiction in the study or intervention, a reviewer searched the acknowledgements for funding agency and methods for direct public health involvement. In addition, the reviewers included any information they believed might have introduced bias into the study. Note that to date, this study protocol has not been registered.

## Results

### Study Selection

**Fig 1:**
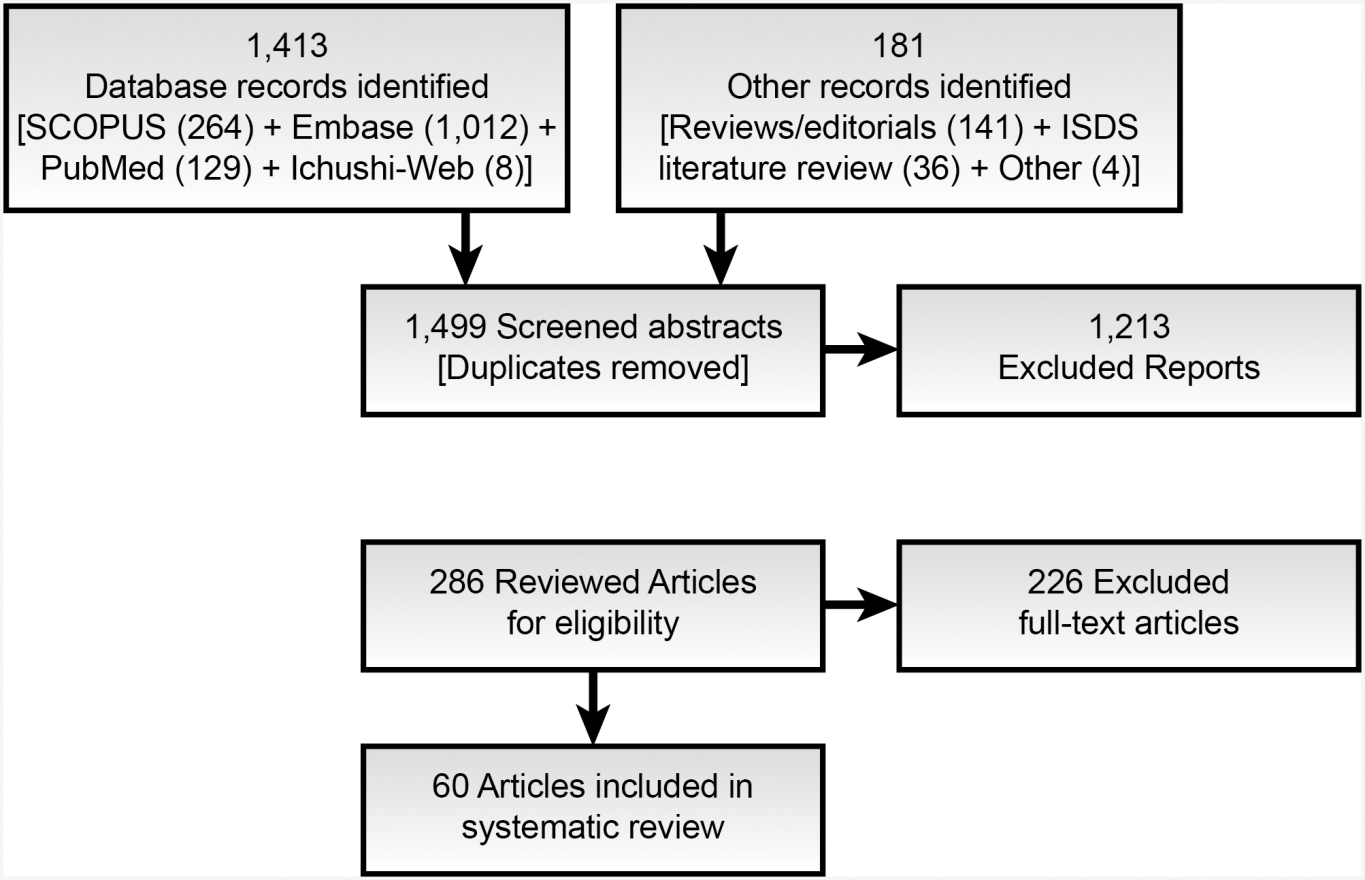
Flow diagram for the selection of literature reviewed. The abstract screening process resulted in 286 studies identified for detailed review of full-text articles. After this review, we further excluded studies that did not meet our definition of social media (e.g., Internet search, ProMED-mail) or discussed methods exclusively. We identified a total of 60 studies that met our eligibility criteria and addressed at least one of the two research questions. This process took over a year to complete because the authors donated their free time to review and analyze the literature.

We identified 1,405 English language studies published through February 2013 in peer-reviewed journals, conference proceedings, and white/gray literature through Embase, PubMed, and Scopus database searches, as well as 8 articles through the Japanese database, Ichushi-Web (Fig 1). An additional 181 studies were identified from citation lists of relevant reviews or editorials and the workgroup’s private collections. After removing duplicates, 1,499 studies remained for the abstract screening step. We excluded 1,205 of these studies because they were reviews, letters, commentaries, or did not address either of the research questions. An additional 8 studies were excluded because the full-text publications were not available. These excluded study abstracts or presentations reported promising preliminary research addressing active disease surveillance. Topics ranged from targeting sexually transmitted diseases in traditionally hard-to-reach populations [10–13] to detecting unusual events, anomalies, and social disruption for early warning systems [14].

### Study Characteristics

#### Question 1 –Intervention into Surveillance Practice and Outbreak Management

Of the 33 studies concerning disease surveillance and outbreak management, 48% (n = 16) were conducted in North America [15–30], 24% (n = 8) in Europe [31–37], 15% (n = 5) in Asia [38–42], 9% (n = 3) in an unspecified location [43–45], 3% (n = 1) in South America [46], and 3% (n = 1) on a global scale [47]. Twitter was used as the primary data source in 81% (n = 27) of the studies although Facebook, various blogs, and health-related discussion forums were also investigated [15–17,19–23,26,27,29–40,43–47]. The studies examined data from January 2006 [46] to January 2012 [38] with the majority focused on 2009. The collection period spanned the 2009 H1N1 influenza pandemic, and 45% (n = 15) of the papers focused on influenza monitoring [15,16,19,27,31–33,38–40,43–45,47]. Comparison to CDC reports were most commonly used to evaluate the effectiveness of the various surveillance techniques presented. Most Twitter-based studies identified study populations through automatic means, i.e., Twitter keyword searches such as "influenza," "H1N1," and "swine flu” to target influenza-related tweets. The articles reported that study sizes were measured either by the number of tweets, ranging from 150 thousand [38] to 2 billion [22], or the number of unique social media users, ranging from 118 users [28] to 24.5 million [33]. Most studies were published in English (2 in Japanese), and all were exploratory in nature.

#### Question 2 –Targeted Vulnerable Populations

**Fig 2:**
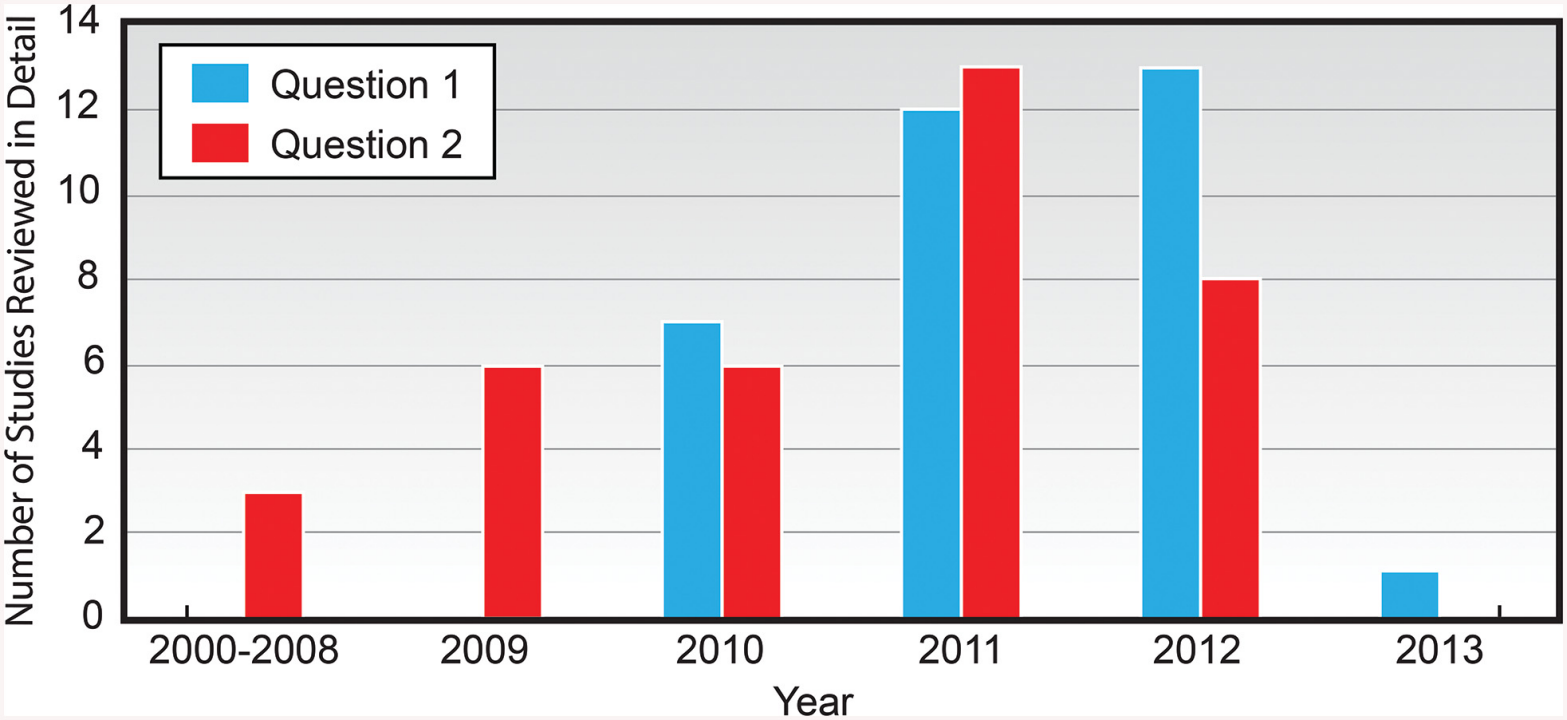
Number of Studies Addressing Questions 1 and 2, Reviewed in Detail by Year Published (January 2000 –February 2013).

Thirty-two studies were identified as targeting a vulnerable population to improve health outcomes. These studies emphasized interaction with users rather than automatic algorithms and therefore typically contained focused populations and smaller datasets. The study sizes ranged from 19 post-partum women [48] to 155,508 Twitter users from 9 distinct areas [26]. All of the studies included were published in English and 66% (n = 21) were conducted in North America [20,21,25,26,49–65], 12% (n = 4) in Australia [48,66–68], 9% (n = 3) in Asia [41,69,70], and 6% (n = 2) in Europe [1,71]. Most of the studies were classified as exploratory, although 24% (n = 8) of studies did include some type of intervention [1,55,56,58,60,64,69,71]. Populations studied were generally more focused than Question 1 studies, e.g., pregnant smokers in Australia. The study populations dated from January 2000 [1] to February 2012 [66], although many do not disclose study periods. Interestingly, the studies addressing Question 2 first appeared in 2000, but published literature on Question 1 does not appear until 2010. Also, there is a spike in addressing both questions during 2011 (Fig 2).

### Bias Across and Within Studies

The spectrum of studies selected for review were subject to publication bias because only primary literature was included and, therefore, other non-published information collected by state or federal health agencies was not incorporated. The choice of data search engines may have excluded valid studies that may not have been published in journals exposed through this process. In addition, there may be more recent articles published since our collection end date of March 2013.

Within the 60 studies reviewed, no important bias was identified by the authors and workgroup reviewers in 43% (n = 26) of the studies [1,19–21,23,24,29,31,34–36,38,39,41,43–45,49,53,57,61–63,66,70,72]; 56% (n = 34) displayed some degree of bias risk. The types of bias can be broken down into six different categories. Selection bias (n = 17) was the most prevalent as data was often collected out of convenience [25,51,52,60], at focused locations [15,46], or within specific social groups [65] and, therefore, was often not representative of the total population [15,26,28,30,46–48,51,54,58,59,65,71,73]. There were 14 articles displaying a faulty study design due to the choice of time period [16,17,27,33,37,40,42,47], data source [18,42,60], study scope [37,42], reporting of results [37,54], or lack of result measure [50,51]. Temporal relationship and directionality bias within 8 studies caused issues in the ability to extrapolate data [16,65,67] or infer directionality or causality [28,32,55,69]. A few studies had sample sizes that were too small to draw conclusions, i.e., sample size bias [17,30,55,64,67]. Other biases present in the reviewed articles were reliability bias [22,28,54,68] and selective interpretation bias [32,40].

### Public Health Involvement

There was a small number of local (3%, n = 2) and state (15%, n = 9) governmental public health agencies involved in the studies reviewed for actionable health and disease surveillance (Table 2). These supportive agencies reside in England (London) and the United States of America (USA) (California, Louisiana, Michigan, and Washington State). Public health involvement was mainly in monetary support on a national level (n = 30) from Brazil, Canada, Germany, Japan, Netherlands, Switzerland, Taiwan, and USA. One research paper, funded by the University of Maryland, USA, described the implementation of social media communication by a local Taiwan government for disaster management, which showed promise over current national awareness and response protocols [69]. Other universities showing interest in support of social media research were located in Australia, Germany, Italy, Japan, United Kingdom (UK), and USA. The private funding agencies that supported reviewed literature are found in the UK and USA. Only 3 papers contained a co-author who was affiliated with a public health agency, i.e., Public Health Agency of Canada [24], Governmental Institute of Public Health of Lower Saxony in Germany [37], and National Cancer Institute in USA [60].

**Table 2:** Counts of Funding Agency Types and Their Direct Involvement in the Review Studies.

	Funding Agency	Funding Agency	Funding Agency	Direct Involvement	Direct Involvement	Direct Involvement
	Question 1	Question 2	Total	Question 1	Question 2	Total
Private	3	5	8	0	0	0
University	8	12	20	0	0	0
Local	1	1	2	0	1	1
State	5	4	9	0	0	0
National	20	10	30	0	0	0
Total	37	32	69	0	1	1

For both research questions, this table records the number of articles in which private organizations, universities, and governments (local, state and national) contributed as funding agencies and/or organizations with direct involvement in each study or intervention reviewed. Note that some articles contained more than one funding agency.

#### Question 1: Can social media be integrated into disease surveillance practice and outbreak management to support and improve public health?

The key to our systematic literature review of Question 1 was to identify if, when, and how social media have been applied for disease surveillance and outbreak management to support and improve public health. Within the 33 manuscripts identified as addressing this question, we found an overwhelming number focused on influenza-like illnesses (45%, n = 15). For the remaining articles, we classified the instances into Infectious Diseases (n = 6), Non-infectious Diseases (n = 4), Medication and Vaccines (n = 3), and Other (n = 5) to understand the extent and focus of current research. All of these studies were exploratory research and did not contain any type of intervention analysis.

#### Influenza-like Illness

Influenza-like illness (ILI) was the first disease to be modeled using social media data in our review (Table 3). We identified 15 original, exploratory studies on ILI targeting social media users (e.g., Twitter and other blogs) from the USA, UK, and Japan between 2008 and 2012. From simple text searches, (e.g., flu or influenza [32,40,45]) to more specific influenza subtypes (e.g., H1N1, Swine Flu [16,44,47]) and symptomatic disease sets [15,17,19,31,32,38,39], all of the studies claimed to be able to use the social media data in real-time disease surveillance. A study by Sadilek, Kautz, and Silenzio (2012), applied their technique to identify the health of any person through geo-tagged Twitter microblogs in an effort to predict disease transmission [15]. In general, correlation between social media data and national health statistics, e.g., from the CDC, ranged from 0.55 [18] to 0.95 [43] and was shown to predict outbreaks before the standard outbreak surveillance method favored by each country [19,31,32,38,39].

**Table 3:** Characteristics of 15 influenza-like illness studies addressing research question 1^a^.

Author (et al.)	Social Media Type	Location	Sample Population	Study Type	Population/Disease Studied	Time Period	Intervention/Exploratory
**Achrekar [** 16 **]**	Twitter	North America	1.9 million Twitter users	Retrospective	Influenza	Not specified	Exploratory
**Aramaki [** 40 **]**	Twitter	Asia	300 million tweets	Retrospective	Influenza (H1N1)	2008–2010	Exploratory
**Chew [** 47]	Twitter	North America	Over 2 million tweets	Retrospective	Influenza	May–Dec 2009	Exploratory
**Collier [** 44 **]**	Twitter	Asia/Europe	5283 tweets	Retrospective	Influenza	2010	Exploratory
**Corley [** 18 **]**	Blogs, forums	North America	158,497,700 English language	Retrospective	Influenza	2008–2009	Exploratory
**Culotta [** 17 **]**	Twitter	North America	574,643 tweets	Retrospective	Influenza	Feb–Apr 2010	Exploratory
**Culotta [** 43 **]**	Twitter	North America	500 million tweets	Other	Influenza	2009/10 flu season	Exploratory
**De Quincey [** 45 **]**	Twitter	Europe	135,000 tweets	Retrospective	Influenza	2009	Exploratory
**Doan [** 33 **]**	Twitter	Europe (UK)	24.5 million Twitter Users	Retrospective	Influenza	2009–2010	Exploratory
**Ishikawa [** 38 **]**	Twitter	Asia (Japan)	157,007 tweets	Prospective cohort	Influenza	July 2011– Jan 2012	Exploratory
**Lampos [** 31 **]**	Twitter	Europe (England & Wales)	200,000 geolocated tweets; Urban centers	Correlation study	Influenza	2009/10 flu season	Exploratory
**Okamura [** 39 **]**	Twitter	Asia (Japan)	716,417 tweets	Retrospective	Influenza	Dec 2010– Jan 2011	Exploratory
**Sadilek [** 15 **]**	Twitter	North America (NYC area)	6237 tweets	Prospective cohort	Influenza	2010	Exploratory
**Signorini [** 19 **]**	Twitter	North America	Twitter users	Retrospective	Influenza (H1N1)	2009	Exploratory
**Szomszor [** 32 **]**	Twitter	Europe	3 million tweets	Retrospective	Influenza (H1N1)	May–Dec 2009	Exploratory

^a^Q1: Can social media be integrated into disease surveillance practice and outbreak detection management to support and improve public health and outbreak management?

#### Infectious Diseases

We identified 6 studies that used different social media programs to determine if the timeliness and sensitivity of detection for other infectious disease outbreaks (e.g., dengue fever, cholera, human immunodeficiency virus (HIV), and *Escherichia coli*) could be improved (Table 4). In a study by Chunara, Andrews, and Brownstein (2013), the volume of cholera-related Twitter posts and HealthMap news media reports were compared to official Haiti cholera case reports during the first 100 days of the 2010 outbreak [23]. The changes in social media and news data trends were detected up to 2 weeks earlier than official case data, which they believe could have had direct implications on the disease outbreak and control measures taken [23]. After analyzing 7 million tweets on medical conditions during the 2011 *Enterohaemorrhagic E*. *coli* (EHEC) outbreak in Germany, Diaz-Aviles et al. (2012) found over 450,000 posts related to the outbreak and determined that this information would have detected the outbreak 1 day earlier than other warning systems [34]. Gomide et al. (2011) showed a correlation between Twitter posts in Brazil and dengue outbreaks (e.g., reported dengue cases correlated with the word “dengue” (0.78) and personal experience with dengue (0.96)) [46]. However, they reported that only 40% of tweets included location, which limited spatial analysis [46]. Although the breadth of studies is limited and most often retrospective, detection of outbreaks through social media tracking appears to provide a timeliness advantage in a variety of infectious disease outbreak settings.

**Table 4:** Characteristics of 6 infectious disease studies addressing research question 1^a^.

Author (et al.)	Social Media Type	Location	Sample Population	Study Type	Population/Disease Studied	Time Period	Intervention/Exploratory
**Stuart Chester [** 24 **]**	Web forums	North America	Mountain biking forum	Cohort	Campylo-bacteriosis outbreak during race	2007	Exploratory
**Chunara [** 23 **]**	Twitter	North America (Haiti)	Twitter data	Not specified	Cholera outbreak	Oct 2010–Jan 2011	Not specified
**Diaz-Aviles [** 34 **]**	Twitter	Europe (Germany)	Twitter data	Case study	*E*. *coli* outbreak	May–Jun 2011	Exploratory
**Diaz-Aviles [** 35 **]**	Twitter	Europe (Germany)	Twitter data	Algorithm development	*E*. *coli* outbreak	May–Jun 2011	Exploratory
**Gomide [** 46 **]**	Twitter	South America (Brazil)	Twitter data	Retrospective	Dengue fever outbreak	2006–2011	Exploratory
**Ku [** 41 **]**	Yahoo forums	Asia (Taiwan)	Yahoo knowledge public health forums	Retrospective	HIV/AIDS content	2007–2009	Exploratory

^a^Q1: Can social media be integrated into disease surveillance practice and outbreak detection management to support and improve public health and outbreak management?

#### Non-infectious Diseases

The 4 studies identified as targeting non-infectious diseases were purely exploratory and focused on alcohol, tobacco, and sexual activity (Table 5). Facebook [25] and Twitter [27] were used to identify associations between alcohol references and misuse in college students or alcohol sales, respectively. It was shown that social media references to alcohol correlated with college students’ self-reported alcohol use, including alcohol-related injuries, and the U.S. Census Bureau’s alcohol sales volume. Therefore, social media data can enhance alcohol use surveillance and target specific audiences in need of health support. Another study, directed at college freshmen’s Facebook use, found a positive correlation between displaying sexual references online and reporting the intention to become sexually active, providing a new forum to target prevention or education messages to adolescents [28]. Prier et al. (2011) examined different tools available to most effectively identify public health topics on Twitter [26]. They found that the Latent Dirichlet Allocation (LDA) topic modeling method was successful in identifying broad topics, e.g., physical activity, obesity, substance abuse, and attitudes towards healthcare, whereas a smaller, more focused dataset created by query selection and theme analysis is necessary to detect lower-frequency topics such as tobacco use. Overall, the study showed that social media can be used to promote both positive and negative heath behaviors.

**Table 5:** Characteristics of 4 non-infectious disease studies addressing research question 1^a^.

Author (et al.)	Social Media Type	Location	Sample Population	Study Type	Population/Disease Studied	Time Period	Intervention/Exploratory
**Culotta [** 27 **]**	Twitter	North America	Twitter data	Retrospective	Influenza rates and alcohol sales volume	2009–2010	Exploratory
**Moreno [** 25 **]**	Facebook	North America	Undergraduates from to U.S. universities	Cross-sectional survey	Problem drinking	2009–2010	Exploratory
**Moreno [** 28 **]**	Facebook	North America	U.S. university students (ages 18 and 19 years)	Cross-sectional	Sexual reference displays	2008–2011	Exploratory
**Prier [** 26 **]**	Twitter	North America	Twitter data	Retrospective	Tobacco use	2010	Exploratory

^a^Q1: Can social media be integrated into disease surveillance practice and outbreak detection management to support and improve public health and outbreak management?

#### Medication and Vaccines

Social media discussions can be used to determine attitudes, misinformation, and adverse events related to medications, vaccines, and other drug uses (Table 6). Salathé and Khandelwal (2011) identified an increase in Twitter data between August and November 2009 related to the launch of the 2009 influenza H1N1 vaccine [20]. Tweets among opinionated users most often shared similar positive or negative sentiments towards vaccine use. As a result, simulation studies of disease transmission result in clusters of individuals with negative vaccine sentiments being unvaccinated and, therefore, at a higher risk of infection. This evidence may assist in targeting public health interventions of unvaccinated people at risk of disease. Another study reported that negative sentiment is more contagious than positive and, therefore, an increase in positive attitudes may predict an even greater increase in negative sentiment, which can be useful in modeling the diffusion of health behavior on social networks [21]. Twitter feeds provide a forum for discussions regarding medications and, therefore, can be targeted to improve information dissemination. Bian et al. scanned Twitter feeds for 5 different drugs and found 239 drug users with 27 drug-related adverse event tweets [22]. This study identifies support for pharmacovigilance through social media analysis, especially concerning new drug releases.

**Table 6:** Characteristics of 4 medicine or vaccine studies addressing research question 1^a^.

Author (et al.)	Social Media Type	Location	Sample Population	Study Type	Population/Disease Studied	Time Period	Intervention/Exploratory
**Bian [** 22 **]**	Twitter	North America	2 billion tweets	Retrospective	Adverse drug events	May 2009 –Oct 2010	Exploratory
**Salathé [** 20 **]**	Twitter	North America	Twitter data	Prospective cohort	Sentiment towards new vaccine	Aug 2009 –Feb 2010	Exploratory
**Salathé [** 21 **]**	Twitter	North America	Twitter data	Prospective cohort	Sentiment towards new vaccine	Aug 2009 –Feb 2010	Exploratory

^a^Q1: Can social media be integrated into disease surveillance practice and outbreak detection management to support and improve public health and outbreak management?

#### Other

Many researchers have evaluated ways to best access and use health information on Twitter for disease surveillance (Table 7). A group in Germany retrospectively reviewed tweets that contained keywords of infectious disease symptoms and found 51% contained headlines that were linked to news websites regarding outbreaks and determined that a potential exists for using Twitter for real-time disease surveillance [37]. Sofean and Smith (2012) designed and evaluated a real-time architecture for collecting and filtering disease-related postings on Twitter and found they could track health status in real time [36]. Other researchers developed methods for pulling social media, including using a Badu search engine [42] and the Ailment Topic Aspect Model (ATAM) [29]. ATAM introduces prior knowledge into the model from articles on diseases, reports model behavior in new settings, tracks illnesses over time and location, correlates risk factors with ailments, and then analyzes the correlations of symptoms and treatments. The ATAM is able to discover any coherent ailments, symptoms and treatment and does not have to be disease-specific [30]. Using a variety of search engines and new tools, it is possible to detect and track a variety of health ailments using social media.

**Table 7:** Characteristics of 5 “other” topic studies addressing research question 1^a^.

Author (et al.)	Social Media Type	Location	Sample Population	Study Type	Population/Disease Studied	Time Period	Intervention/Exploratory
**Krieck [** 37 **]**	Twitter	Europe (Germany)	Twitter data	Retrospective	Fever, swine flu & H1NI-related	Sep 2010 –Feb 2011	Exploratory
**Paul [** 30 **]**	Twitter	North America	2 billion tweets	Not specified	Not specified	2010	Exploratory
**Paul [** 29 **]**	Twitter	North America	2 billion tweets	Not specified	Not specified	2010	Explanatory
**Sofean [** 36 **]**	Twitter	Europe	Twitter data	Algorithm development	Twitter data	Not specified	Exploratory
**Yang [** 42 **]**	Weblogs, microblogs, wikis, etc.	Asia	Weblogs, microblogs, wikis, etc.	Case study	Chinese social media data	Not specified	Not specified

^a^Q1: Can social media be integrated into disease surveillance practice and outbreak detection management to support and improve public health and outbreak management?

### Question 2: Can social media be used to effectively target populations, specifically vulnerable populations, to test an intervention and interact with a community to improve health outcomes?

For question 2, we identified if, when, and how social media have been used to target populations and transform information gleaned from this data into action. The majority of studies within this group used social media to identify health risk behaviors (n = 10) and evaluate use of virtual communities to aid in risk reduction. For the remaining articles, we classified the instances into Infectious Diseases (n = 3), Non-infectious Diseases (n = 9), and Other (n = 10) to get a better overview where exploratory research (n = 25) and intervention efforts (n = 7) have been focused.

#### Health Risk Behaviors

Social media, especially Facebook [25,49,68] and MySpace [56,57], have been used to target adolescents displaying health risk behaviors associated with substance abuse and sexual activities (Table 8). Specialized chat rooms, websites, and Twitter have been targeted for adult health risk behavior with tobacco use [26,48,60], substance abuse [53], and sexual activities [58]. The specific populations, located in the USA and Australia, include college students [49,56,68], post-partum women [48], men who have sex with men (MSM) [58], and low-income youth [57]. These studies show that social media can be effective at identifying adolescent populations displaying substance abuse, especially alcohol [25,49,68], in addition to sexual behavior [57], and that social media can improve community health outcomes in at-risk adolescents [56] and MSM [58]. Interestingly, tobacco-related subjects posed an issue for researchers who tried to use topic modeling in Twitter [26] and found that the use of a virtual community bulletin board to reduce smoking behavior was ineffective [60]. As proposed by Prier et al. (2011), the use of low-frequency topics, such as tobacco use, may require human intervention for selection of query terms and relevant subsequent analysis to properly address health concerns [26].

**Table 8:** Characteristics of 10 health risk behavior studies addressing research question 2^a^.

Author (et al.)	Social Media Type	Location	Sample Population	Study Type	Population/Disease Studied	Time Period	Intervention/Exploratory
**Frost [** 53 **]**	PatientsLikeMe	North America (USA)	Patient members of PatientsLikeMe	Retrospective	Self-reported outcomes of off-label drug use	2010	Exploratory
**Litt [** 49 **]**	Facebook	North America (DC area)	89 Adolescents (ages 13–15 years); spend at least 1 hour on Facebook/week	Randomized	Perceptions of alcohol	2011	Exploratory
**Lowe [** 48 **]**	Any type of social media	Australia	Women who visited an ante-natal clinic	Prospective cohort	Pregnant smokers	2011	Exploratory
**Moreno [** 25 **]**	Facebook	North America	Undergraduates from 2 U.S. universities	Cross-sectional survey	Problem drinking	2009–2010	Exploratory
**Moreno [** 56 **]**	MySpace	North America	Ages 18–20 years; at least 1 risk behavior (sexual or substance abuse)	Randomized controlled intervention trial	Adolescent risk behavior (sexual and substance)	2007	Intervention
**Prier [** 26 **]**	Twitter	North America	Twitter data	Retrospective	Tobacco use	2010	Exploratory
**Ralph [** 57 **]**	TeenSMART and MySpace	North America (California)	Teens in lower-income areas	Survey, interviews, focus groups	General sexual health	July–Sept 2008	Exploratory
**Rhodes [** 58 **]**	Chat room	North America (North Carolina)	1,851 users; 210 users online assessment	Trained interventions and questionnaire	HIV risk behaviors for MSM	2004–2005	Both
**Ridout [** 68 **]**	Facebook	Australia	158 university students	Retrospective	Alcohol use	2009–2010	Exploratory
**Stoddard [** 60 **]**	Web-based Bulletin board	North America (USA)	1375 adult federal employee or contractors	Randomized	Willing to quit smoking	2006–2007	Intervention

^a^Q2: Can social media be used to effectively target populations, specifically vulnerable populations, to test an intervention and interact with a community to improve health outcomes?

#### Infectious Diseases

Two of the 3 social media studies focusing on infectious diseases (67%), investigated the use of social media to reach target populations for protection against sexually transmitted infections (STI) (Table 9). For example, Sullivan et al. (2011) identified factors behind the underrepresentation of black and Hispanic MSM in online research studies (ORS) despite this group experiencing the largest increase in HIV case reports [62]. Targeted banner advertisements were posted in MySpace, displaying an ethnicity-matched model. This approach increased the odds of click-through of the ORS (adjusted odds ratios 1.7–1.8), but with limited effect on reducing dropouts. In the 2009 H1N1 pandemic, Szomszor, Kostkova, and de Quincey found that health communication via official Twitter feeds and trusted news organizations (e.g., BBC) was most effective in reaching the public; however, timeliness of health information may not directly translate to site popularity among these trusted sources [72]. In addition, they found 40% of appreciable health-related information identified on the Internet containing poor scientific merit was directly linked to spam. Overall, the studies showed potential in reaching populations concerning socially stigmatized or sensitive health conditions, but time and effort are needed to build up a trusted channel for information dissemination.

**Table 9:** Characteristics of 3 infectious diseases studies addressing research question 2^a^.

Author (et al.)	Social Media Type	Location	Sample Population	Study Type	Population/Disease Studied	Time Period	Intervention/Exploratory
**Ku [** 41 **]**	Yahoo forums	Asia (Taiwan)	Yahoo knowledge public health forums	Retrospective	HIV/AIDS content	2007–2009	Exploratory
**Sullivan [** 62 **]**	MySpace banner ad	North America (USA)	MySpace MSM	Online survey	MSM at risk for HIV	2009	Exploratory
**Szomszor [** 72 **]**	Twitter	Global	English language users	Retrospective	Swine flu outbreak	2009	Exploratory

^a^Q2: Can social media be used to effectively target populations, specifically vulnerable populations, to test an intervention and interact with a community to improve health outcomes?

#### Non-Infectious Diseases

Social media could potentially be used to target populations with illnesses of high prevalence and public health impact (e.g, depression, cancer, obesity, diabetes, and asthma) with an intervention to improve health outcomes. In a 16-week study of 32 women with breast cancer, an intervention using an electronic support group reported a significant decrease in depression symptoms and reaction to pain, and a trend towards increasing posttraumatic growth, zest for life, and deepening of spiritual lives [55]. There were some dropouts in participation, which was attributed to different personalities’ response to the electronic support group. Similarly, researchers set up a chat room to provide an educational tool for adolescents with Type 1 diabetes and found that it significantly increased compliance and decreased HbA(1c) concentrations (from 8.9% to 7.8%) over a period of 3 months [1]. Mobile support programs used to increase dietary self-monitoring and improve weight loss resulted in body weight changes; however, a similar study using Twitter did not find any differences [64]. Therefore, the types of social media and the populations who will use and benefit from this type of information are key factors in how they impact health.

Multiple studies attempted to determine whether the potential exists for social media to reach vulnerable populations (Table 10). For mental health, a study of college freshmen showed that 46% of female and 21% of male students referenced stress, depression, or stress-related conditions, e.g., weight issues or drinking alcohol, on Facebook, and those who referred to stress were significantly more likely to mention weight concerns or depression [51]. These researchers concluded that Facebook may provide a mode of distribution of targeted stress reduction information. Similarly, researchers in Australia found that 44% of students reported the need for mental health support; within this group, 50% of them already use the Internet and 47% said they would use online social networks for mental health problems [67]. Social media could be used to identify those with non-infectious diseases and provide education and support to improve public health.

**Table 10:** Characteristics of 9 non-infectious diseases studies addressing research question 2^a^.

Author (et al.)	Social Media Type	Location	Sample Population	Study Type	Population/ Disease Studied	Time Period	Intervention/Exploratory
**Baptist [** 50 **]**	Facebook, MySpace, Twitter, etc.	North America	Asthma patients, ages 12–40 years	Cross-sectional	Asthma	2010	Exploratory
**Egan [** 51 **]**	Facebook	North America	300 U.S. university students, ages 18–19 years	Prospective cohort	Stress-related conditions	2009–2010	Not specified
**Iafusco [** 1 **]**	Chatline	Europe (Italy)	Young people with type 1 diabetes	Not specified	Diabetes care	2000	Intervention
**Lieberman [** 55 **]**	Discussion forums	North America	32 women with breast carcinoma	Clinical trial	Benefits of discussion forum for breast cancer	Not specified	Intervention
**O’Dea [** 67 **]**	MySpace, Facebook, others	Australia	Convenience sample of rural Australian high school students	Mixed methods	Social media for mental health	Not specified	Exploratory
**Song [** 59 **]**	Other (social networking site)	North America (Los Angeles, CA)	14 childhood cancer survivors (ages 18–25 years)	Content analysis (video clips)	Quality of life issues for childhood cancer survivors	Not specified	Exploratory
**Takahashi [** 70 **]**	Japanese social network	Asia	105 participants	Observational cross-section study	Depression-oriented social networking site	2007	Exploratory
**Tsaousides [** 63 **]**	Facebook	North America	96 individuals (60% female, ages 23–70 years) with traumatic brain injuries	Qualitative (online survey)	Use among individuals with traumatic brain injuries	Not specified	Exploratory
**Turner-McGrievy [** 64 **]**	Mobile Pounds Off Digitally (POD)	North America (USA)	Men and women users of POD	Randomized	Overweight and obese adults	July 2010 –Feb 2011	Intervention

^a^Q2: Can social media be used to effectively target populations, specifically vulnerable populations, to test an intervention and interact with a community to improve health outcomes?

#### Other

Social media was used to identify “other” target populations, e.g., low-income groups [61] and older people in need of physical activity [71], to assess vaccination sentiments [20] and misuse of antibiotics [73] (Table 11). Salathé et al. (2011) found a strong correlation (r = 0.78) between vaccination sentiments on Twitter and vaccination rates reported by the CDC across U.S. Department of Health and Human Service regions [21]. Clusters of unprotected individuals with negative vaccination sentiments can be identified and targeted for tailored interventions. Scanfeld, Scanfeld, and Larson (2010) identified individuals from Twitter who may have misused antibiotics for treating viral infections who could be targeted for health-related education [73]. Dissemination of valid health information among the identified groups may promote behavioral change towards a healthier lifestyle.

**Table 11:** Characteristics of 10 “other” topic studies addressing research question 2^a^.

Author (et al.)	Social Media Type	Location	Sample Population	Study Type	Population/ Disease Studied	Time Period	Intervention/ Exploratory
**Dumbrell [** 66 **]**	Twitter	Australia	Tweets from 114 health organizations	Case study	Various public health issues	2012	Exploratory
**Fisher [** 52 **]**	Various	North America (Utah)	111 patients at outpatient facility	Survey	Interests of patients in social media	2011	Exploratory
**Huang [** 69 **]**	Plurk	Asia (Taiwan)	>24,000 people displaced after extreme weather event	Case study	Locate displaced people after extreme weather	Aug 2009	Exploratory
**Idriss [** 54 **]**	Forum for psoriasis	North America	260 subjects from 5 online psoriasis support groups	Case study (online survey)	Demo-graphics, usage patterns and experiences	2006–2007	Exploratory
**Peels [** 71 **]**	Discussion forums	Europe (Netherlands)	> 7000 participants over 50 years old	Mixed methods	Increasing physical activities	2005–2010	Intervention
**Salathé [** 20 **]**	Twitter	North America	Twitter data	Prospective cohort	Sentiment towards new vaccine	Aug 2009 –Feb 2010	Exploratory
**Salathé [** 21 **]**	Twitter	North America	Twitter data	Prospective cohort	Sentiment towards new vaccine	Aug 2009 –Feb 2010	Exploratory
**Scanfield [** 73 **]**	Twitter	North America	Twitter data	Content analysis	Antibiotic information	2009	Exploratory
**Stroever [** 61 **]**	Facebook	North America	Low-income parents	Qualitative (focus group)	Value of child health information to low-income parents	2010	Exploratory
**Wicks [** 65 **]**	PatientsLikeMe	North America (80% USA)	7000 PatientsLikeMe members	Cross-sectional study	PatientsLikeMe Usefulness	2009	Exploratory

^a^Q2: Can social media be used to effectively target populations, specifically vulnerable populations, to test an intervention and interact with a community to improve health outcomes?

## Discussion

This systematic primary literature review on the use of social media to support public health practice has identified many evidence gaps and biases in the current knowledge on this topic. There are few studies to date on interventions and a lack of use of social media in practice despite the high potential for success identified in exploratory studies. This mirrors the lack of scientific reports published (n = 16) on performance assessment of disease surveillance methods found by Babaie et al. (2015), regardless of their necessity to public health response [74]. Our findings may suggest that it is particularly challenging to translate research using social media for biosurveillance into practice. This challenge may be amplified by the lack of an ethical framework for the integration of social media into public health surveillance systems [75]. In addition, the focus of many studies, especially on infectious diseases, is done retrospectively, potentially highlighting the ease in prediction post outbreak rather than implementation of social media prospectively. The under-representation of social media analytics in active surveillance may be due to a lack of resources or technical skills necessary for successful execution in the public health domain. Alternatively, public health departments may be using social media as a tool but not publishing their efforts. Due to the number of heterogeneous data sources used in analysis, a comparison and evaluation of techniques was not possible. However, this review demonstrates some evidence that the use of social media data could provide real-time surveillance of health issues, speed up outbreak management, and identify target populations necessary to support and improve public health and intervention outcomes.

Social media can impact the public health surveillance domain, bringing the wider media landscape to the public health community. This impact has been particularly important in the context of public health emergencies, such as after Haiti's post-earthquake cholera outbreak, where the utility of using social media as a data source in rapidly changing and dynamic situations was clearly shown [23]. Pharmacovigilance is another key area where social media have demonstrated value. Traditional methods of reporting adverse drug events rely on gatekeepers (e.g., clinicians and pharmaceutical companies) to alert authorities of these events. Social media, in particular Twitter, have shown significant potential for creating real-time access to firsthand reports of adverse drug events, thereby bypassing the gatekeeper bottleneck [22,76].

Traditionally hard-to-reach groups, e.g., MSM and adolescents, may be more likely to engage with social media rather than with more conventional public health communication channels, creating a new avenue to address sensitive health issues. A significant proportion of the interventions reviewed (40%) concentrated on targeting populations with increased risk of STIs, a topic often avoided in public settings [11,12,56,58]. Mental health intervention studies suggested that young people would be willing to use social media to address mental health issues [51,65,67,70]. In this context, the type of mediations must fit the social media outlet targeted. For example, mental health interventions conducted via Twitter, with a 140 character limit, are likely to be very different from the kinds of interventions conducted through the more discursive communication possible with Internet discussion forums.

Different target groups, e.g., age groups, may prefer different social media outlets. Consequently, knowing the population and how they use social media can be a critical part of successful intervention and surveillance. For example, in the articles reviewed focusing on health risk behaviors, we found that adolescents were targeted using Facebook [25,49,68] and MySpace [10,56,57], while adults were targeted within Twitter and specialized chat rooms and websites [26,48,53,58,60]. However, to our knowledge, there is no directed health-related scientific research addressing which social media outlet should be targeted for specific populations. For health surveillance, the impact of the potential lack of population representativeness in the use of social media to detect and track disease outbreaks has not been adequately researched.

In addition, different topics may require different search techniques to identify targeted populations. In the study by Prier et al. (2011), they were successful at identifying broad topics in social media, e.g., physical activity and obesity, using the LDA method, yet for lower-frequency topics, such as tobacco use, human intervention was required for selection of query terms to create a smaller, more focused data set in which subsequent analysis was possible [26]. Similarly, the type of social media platform used in analyses may change based on the target population and the years being studied. For example, the use of social media has evolved from blogs and discussion forums pre–2005 to social networking platforms as new technologies came to market. In this respect, the field of surveillance would benefit from a study classifying topics of concern and appropriate analysis techniques to achieve the greatest number of results or largest audience for intervention.

Since February 2013, the last search date reported in this review, there has been an increase in the number of articles published per year using a similar initial search query to this review. Estimating an 80% exclusion rate based on the results discussed above, around 90 new articles per year were published in 2013 and 2014. This number is higher than previous years, although many of the new literature focus is on previously described sources (e.g., google flu) and methods being applied to a different disease. Regardless, an upward trend in publications suggests an increase interest in understanding social media’s role in disease surveillance. This literature review demonstrates the effectiveness of social media in supporting and improving public health and identifying target populations for intervention. Coupled with the increased interest in social media analytics, opportunities to integrate this novel data source into disease surveillance and outbreak management should arise for public health professionals.

## Supporting Information

S1 Checklist PRISMA checklist for systematic review process. (DOC)
